# Differential Longevity of Memory CD4 and CD8 T Cells in a Cohort of the Mothers With a History of ZIKV Infection and Their Children

**DOI:** 10.3389/fimmu.2021.610456

**Published:** 2021-02-12

**Authors:** Jessica Badolato-Corrêa, Fabiana Rabe Carvalho, Iury Amancio Paiva, Débora Familiar-Macedo, Helver Gonçalves Dias, Alex Pauvolid-Corrêa, Caroline Fernandes-Santos, Monique da Rocha Queiroz Lima, Mariana Gandini, Andréa Alice Silva, Silvia Maria Baeta Cavalcanti, Solange Artimos de Oliveira, Renata Artimos de Oliveira Vianna, Elzinandes Leal de Azeredo, Claudete Aparecida Araújo Cardoso, Alba Grifoni, Alessandro Sette, Daniela Weiskopf, Luzia Maria de-Oliveira-Pinto

**Affiliations:** ^1^Laboratory of Viral Immunology, Fundação Oswaldo Cruz, Rio de Janeiro, Brazil; ^2^Multiuser Laboratory for Research in Nephrology and Medical Science, School of Medicine, Universidade Federal Fluminense, Niterói, Brazil; ^3^Department of Veterinary Integrative Biosciences, Texas A&M University, College Station, TX, United States; ^4^Laboratory of Respiratory Viruses and Measles, Fiocruz, Rio de Janeiro, Brazil; ^5^Laboratory of Cellular Microbiology, Fundação Oswaldo Cruz, Rio de Janeiro, Brazil; ^6^Laboratory of Virological Diagnosis, Biomedical Institute, Universidade Federal Fluminense, Niterói, Brazil; ^7^Department of Maternal and Child, School of Medicine, Universidade Federal Fluminense, Niterói, Brazil; ^8^Center for Infectious Disease and Vaccine Research, La Jolla Institute for Immunology (LJI), San Diego, CA, United States; ^9^Division of Infectious Diseases and Global Public Health, Department of Medicine, University of California, San Diego, San Diego, CA, United States

**Keywords:** Zika, T cells, memory, pregnancy, congenital Zika syndrome (CZS)

## Abstract

**Background:** Zika virus (ZIKV) infection causes for mild and self-limiting disease in healthy adults. In newborns, it can occasionally lead to a spectrum of malformations, the congenital Zika syndrome (CZS). Thus, little is known if mothers and babies with a history of ZIKV infection were able to develop long-lasting T-cell immunity. To these issues, we measure the prevalence of ZIKV T-cell immunity in a cohort of mothers infected to the ZIKV during pregnancy in the 2016–2017 Zika outbreak, who gave birth to infants affected by neurological complications or asymptomatic ones.

**Results:** Twenty-one mothers and 18 children were tested for IFN-γ ELISpot and T-cell responses for flow cytometry assays in response to CD4 ZIKV and CD8 ZIKV megapools (CD4 ZIKV MP and CD8 ZIKV MP). IFN-γ ELISpot responses to ZIKV MPs showed an increased CD4 and CD8 T-cell responses in mothers compared to children. The degranulation activity and IFN-γ-producing CD4 T cells were detected in most mothers, and children, while in CD8 T-cells, low responses were detected in these study groups. The total Temra T cell subset is enriched for IFN-γ+ CD4 T cells after stimulation of CD4 ZIKV MP.

**Conclusion:** Donors with a history of ZIKV infection demonstrated long-term CD4 T cell immunity to ZIKV CD4 MP. However, the same was not observed in CD8 T cells with the ZIKV CD8 MP. One possibility is that the cytotoxic and pro-inflammatory activities of CD8 T cells are markedly demonstrated in the early stages of infection, but less detected in the disease resolution phase, when the virus has already been eliminated. The responses of mothers' T cells to ZIKV MPs do not appear to be related to their children's clinical outcome. There was also no marked difference in the T cell responses to ZIKV MP between children affected or not with CZS. These data still need to be investigated, including the evaluation of the response of CD8 T cells to other ZIKV peptides.

## Introduction

The emergence of ZIKV in dengue-endemic regions creates a potentially alarming scenario, as those caused by the ZIKV epidemic spread across countries, especially the Americas, during 2015–2016 (1, 2). At present, half of the world's population is considered at risk of dengue virus (DENV), and cases of ZIKV continue to be reported globally (3, 4).

DENV and ZIKV are members of the family *Flaviviridae* and are among the several medically important viruses (5). Both are spread *via* the bite of infected mosquitoes, *Aedes spp*., whose ecological niches expand beyond the tropical and sub-tropical regions (6). Moreover, ZIKV can be transmitted *via* sexual contact (7, 8). It persists for weeks in the reproductive tract (9–11) and undergoes vertical transmission from a mother to fetus (12–15). During Latin America and French Polynesia outbreaks, ZIKV infection typically produces mild symptoms that resolve rapidly. However, when an infection occurs during pregnancy, occasional vertical transmission can lead to a spectrum of devastating neurodevelopmental aberrations, collectively referred to as congenital Zika syndrome (CZS) (16). Conflicting data sets indicate that infants born to mothers infected with ZIKV during pregnancy carry up to 42% risk of developing overt clinical or neuroimaging abnormalities (17–21).

ZIKV is closely related to four serotypes of DENV. They are a positive-sense, single-stranded enveloped RNA virus. The genome encodes a polyprotein, which is processed into three structural proteins [the capsid (C), premembrane (prM), and the envelope (E) protein] and seven non-structural proteins (NS1, NS2A, NS2B, NS3, NS4A, NS4B, and NS5) (22). DENV and ZIKV share 55.1–56.3% amino acid sequence identity (23).

Tonnerre et al. performed a remarkably interesting longitudinal study with samples from 10 non-pregnant women with ZIKV-confirmed acute infection. For the T cell response, the authors confirmed different virus-specific targets for CD4 and CD8 T cells (24). They found that previous DENV infections largely affect the humoral response to ZIKV, with effects on the T cell side limited to increasing the frequency of ZIKV-specific CD8 T cells in some patients (24, 25).

Although viral infections are common during pregnancy, transplacental passage, and fetal infection appear to be the exception rather than the rule. Viral infections during pregnancy have been linked to adverse pregnancy outcomes and birth defects in offspring. Unfortunately, there are limited therapeutic or preventive tools to protect both mother and fetus during pandemics (26).

In the present study, we studied ZIKV memory T cell responses from a cohort of mothers infected with ZIKV during pregnancy in the 2016–2017 Zika outbreak who gave birth to infants affected by neurological complications or asymptomatic ones. These donors with a history of ZIKV infection were evaluated in 2018–2019, 2–3 years after ZIKV infection. This cohort provides a unique opportunity to study ZIKV immunity in an infection occurring during pregnancy and compare it with longitudinal follow-up samples.

## Materials and Methods

### Study Design, Volunteers, and Samples

**Figure 1:**
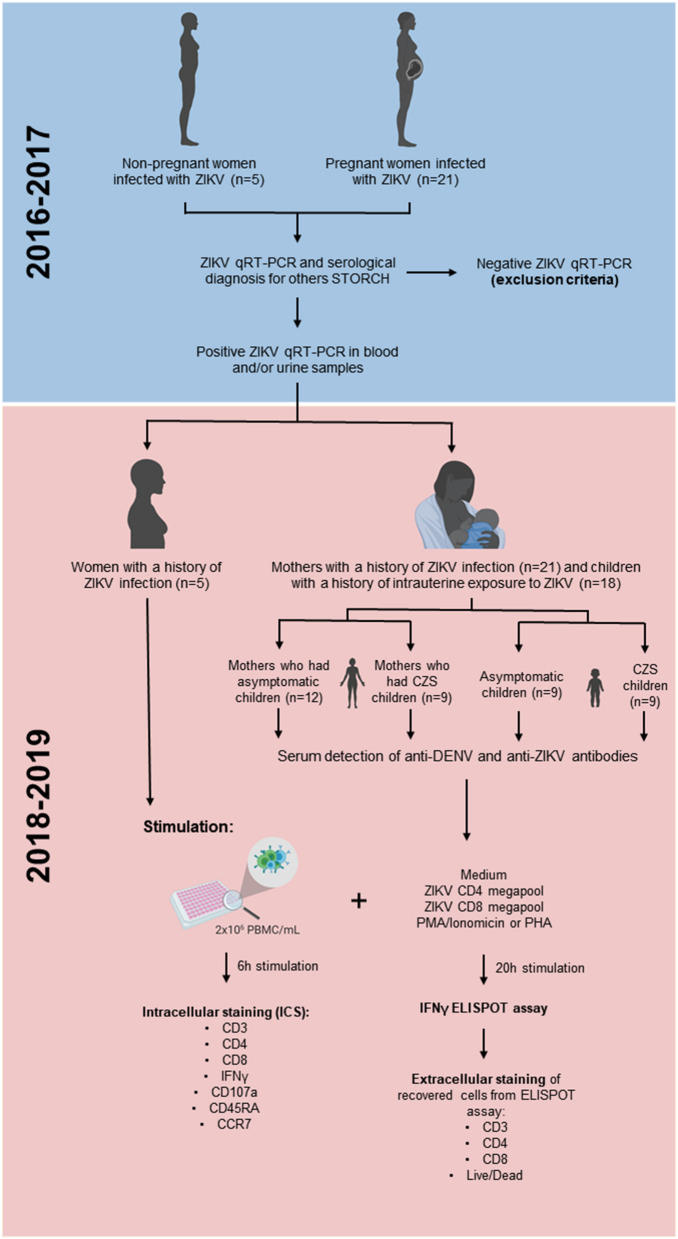
The experimental design used in this study.

A cross-sectional study was carried out in pregnant mothers infected with ZIKV and children born to mothers who reported rash during pregnancy overlapping with the ZIKV Public Health Emergency of National Concern in Brazil period (November 2015 and May 2017). These donors' cohort was referred from the Exanthematic Diseases Unit at the Hospital Universitário Antonio Pedro of the Universidade Federal Fluminense (HUAP/UFF) located in Niteroi, Rio de Janeiro (Brazil). Laboratory evidence for ZIKV infection during pregnancy was based on a mother's positive quantitative real-time (qRT)-PCR test result on serum and/or urine samples, done at the flavivirus reference laboratory of Rio de Janeiro State (LACEN, RJ, Brazil) and confirmed by Multiuser Laboratory for Research Support in Nephrology and Medical Science (LAMAP, UFF, Brazil) (27). A qRT-PCR positive test result at any point after maternal rash onset confirmed the ZIKV infection within the first 5 days of rash and/or the urine sample was tested by the 14th day. So, we included 21 pregnant mothers who presented rash, with or without other clinical symptoms suggestive of arbovirus infections, such as arthralgia, myalgia, fever, headache, and conjunctival hyperemia. The exclusion criteria were mothers who were positive with a qRT-PCR for other arboviruses such as chikungunya and dengue. Moreover, mothers who presented positive test results to syphilis, toxoplasmosis, rubella, cytomegalovirus, and HIV infection were excluded. All serology tests were performed by the Service of Clinical Pathology (HUAP, UFF Brazil) (Figure 1). Three mothers were infected in the first trimester of pregnancy, 14 in the second trimester, 3 in the third trimester, and 1 before pregnancy (Table 1), thus making any laboratory diagnosis of acute Zika infection in babies impossible.

**Table 1:** Characteristics of the recovered mothers infected to Zika virus during pregnancy and their intrauterine exposed children recruited from 2018 to 2019.

**Group**	**Outcome at birth**	**ID**	**Age^**a, b**^**	**Illness time^**b**^**	**Gestational trimester at onset rash**	**State**	**RT-aPCR ZIKV**	**ZIKV anti-IgG**	**DENV anti-IgG**	**PRNT 90 ZIKV**	**PRNT 90 DENV-1**	**CD4+CD3+ T cells**	**CD8+CD3+ T cells**	**CD4/CD8**
Women		W1	40	36		RJ	pos	pos	pos	<10	≥10	77.5	13.8	5.6
		W2	40	40		RJ	pos	pos	pos	<10	<10	74.2	18.0	4.1
		W3	23	38		RJ	pos	pos	pos	<10	≥10	60.1	29.4	2.0
		W4	25	42		RJ	pos	pos	pos	<10	≥10	58.9	22.2	2.7
		W5	27	35		RJ	pos	pos	pos	<10	≥10	76.3	20.0	3.8
			27.0 (23–40)^a^	38.0 (35–42)								69.9 (41.5–76.7)	20.0 (13.8–29.4)	3.8 (2–5.6)
Mothers	Asympt.	M1	22	38	3rd	RJ	pos	pos	neg	160	<10	81.6	9.81	8.3
	Asympt.	M2	36	40	3rd	RJ	pos	pos	pos	>320	≥10	65.2	22.3	2.9
	Asympt.	M3	37	38	2nd	RJ	pos	pos	pos	>320	≥10	62.5	16.5	3.8
	Asympt.	M4	23	24	2nd	RJ	pos	pos	neg	>320	<10	66.6	20.2	3.3
	Asympt.	M5	27	40	2nd	RJ	pos	pos	neg	>320	≥10	71.9	19.5	3.7
	Asympt.	M6	33	38	2nd	RJ	pos	pos	pos	<10	<10	61.0	29.2	2.1
	Asympt.	M7	37	38	2nd	RJ	pos	pos	pos	>320	≥10	73.7	21.1	3.5
	Asympt.	M8	41	x	2nd	RJ	pos	neg	neg	>320	<10	61.6	31.1	2.0
	Asympt.	M9	21	38	2nd	RJ	pos	pos	pos	>320	≥10	72.9	17.1	4.3
	Asympt.	M10	32	40	2nd	RJ	pos	pos	pos	>320	≥10	66.9	20.7	3.2
	Asympt.	M11	30	39	2nd	RJ	pos	pos	pos	>320	≥10	69.5	5.49	12.7
	Asympt.	M12	33	39	2nd	RJ	pos	pos	pos	>320	≥10	66.0	16.7	4.0
			32.5 (21–41)^a^	38.0 (24–40)								66.8 (61–81.6)	19.9 (5.5–31.1)	3.6 (2–12.7)
Mothers	CZS	M13	24	23	1st	RJ	pos	pos	neg	>320	<10	71.6	21.6	3.3
	CZS	M14	42	39	2nd	RJ	pos	pos	pos	>320	<10	67.1	22.5	3.0
	CZS	M15	25	37	3rd	RJ	pos	pos	neg	>320	≥10	66.0	17.4	3.8
	CZS	M16	21	42	1st	RJ	pos	pos	pos	>320	≥10	57.4	24.3	2.4
	CZS	M17	40	40	2nd	RJ	pos	pos	pos	>320	≥10	76.5	17.7	4.3
	CZS	M18	41	36	2nd	RJ	pos	pos	pos	>320	<10	71.3	22.3	3.2
	CZS	M19	28	38	2nd	RJ	pos	pos	pos	>320	≥10	80.5	13.9	5.8
	CZS	M20	21	39	before	RJ	pos	pos	pos	>320	<10	62.7	23.2	2.7
	CZS	M21	28	41	1st	RJ	pos	neg	pos	>320	≥10	78.9	14.9	5.3
			28.0 (21–42)^a^	39.0 (23–42)								71.3 (57.4–80.5)	21.6 (13.9–24.3)	3.3 (2.4–5.8)
Children	Asympt.	C1	35	36		RJ	x	neg	neg	x	x	56.1	29.2	1.9
	Asympt.	C2	29	38		RJ	x	neg	neg	<10	≥10	63.9	25.3	2.5
	Asympt.	C3	31	38		RJ	x	neg	pos	x	x	59.2	17.3	3.4
	Asympt.	C4	34	37		RJ	x	pos	pos	>320	≥10	80.0	15.2	5.3
	Asympt.	C5	41	39		RJ	x	neg	neg	<10	≥10	70.9	19.4	3.7
	Asympt.	C6	32	38		RJ	x	neg	neg	<10	<10	54.7	30.7	1.8
	Asympt.	C7	38	37		RJ	x	neg	neg	<10	<10	53.5	35.5	1.5
	Asympt.	C8	24	39		RJ	x	neg	neg	<10	≥10	72.6	15.3	4.7
	Asympt.	C9	x	x		RJ	x	neg	neg	<10	≥10	74.1	8.12	9.1
			33.0 (24–41)^b^	38 (24–39)								67.4 (53.5–80.2)	18.4 (14.2–35.5)	3.5 (1.5–5.4)
Children	CZS	C10	29	46		RJ	x	neg	neg	<10	≥10	41.5	31.3	1.3
	CZS	C11	17	26		RJ	x	neg	pos	<10	≥10	58.8	32.9	1.8
	CZS	C12	22	22		RJ	x	neg	pos	160	≥10	80.3	11.7	6.9
	CZS	C13	30	30		RJ	x	x	x	x	x	74.8	11.7	6.4
	CZS	C14	23	38		RJ	x	neg	neg	<10	≥10	76.9	7.37	10.4
	CZS	C15	24	40		RJ	x	neg	neg	<10	≥10	50.9	35.1	1.5
	CZS	C16	32	35		RJ	x	neg	pos	<10	<10	76.5	11.3	6.8
	CZS	C17	32	43		RJ	x	neg	pos	<10	≥10	64.1	29.8	2.2
	CZS	C18	29	30		RJ	x	pos	pos	<10	<10	69.9	18.6	3.8
			29.0 (17–32)^b^	36.5 (22–46)								69.9 (41.5–80.3)	18.6 (7.4–35.1)	3.8 (1.3–10.4)

Age^a^, years.

Age^b^, months.

Illness time^b^, months.

*median (minimum–maximum)^a^*.

Eighteen children aged 17–38 months with a history of intrauterine exposure to ZIKV were classified as asymptomatic or with CZS if the test was negative for other congenital infections. The asymptomatic ZIKV group (positive maternal qRT-PCR, *n* = 9) consisted of patients with maternal exposure to ZIKV during pregnancy and no clinical evidence of CZS. The CZS ZIKV group (positive maternal qRT-PCR, *n* = 9) consisted of patients with exposure to ZIKV during pregnancy and with clinical evidence of CZS. Under both ZIKV conditions, mothers had negative results for other infectious agents (syphilis, toxoplasmosis, rubella, cytomegalovirus, and HIV). All participants were clinically evaluated by a multidisciplinary team and are included in an ongoing clinical follow-up program (28). Neuroimaging, such as skull ultrasound, CT or MRI, was performed to investigate radiological factors compatible with congenital infectious diseases. In symptomatic children, according to the Ministry of Health of Brazil, the presence of CZS is defined by maternal ZIKV infection confirmed through qRT-PCR, with two or more clinical parameters, such as microcephaly or other neurological changes; visual or auditory anomalies; and functional disorders, such as irritability, dysphagia, and spasms (29). However, even if the mother has compatible symptoms and is qRT-PCR ZIKV+, it is not possible to state what exposure children who were born asymptomatic had (Figure 1).

From 2018 to 2019, blood donations from these donors with a history of ZIKV infection were collected at HUAP/UFF to be used in our study. Previous exposure to DENV or ZIKV were determined by the presence of detectable DENV-specific immunoglobulin G (IgG) titers (30) or ZIKV-specific IgG titers (31) (Table 1).

As a control group, we had 5 non-pregnant women infected with ZIKV in the same timeframe (2015–2017). These controls were invited to participate in this study because they had confirmed the diagnosis of ZIKV by qRT-PCR and had positive anti-ZIKV IgG serology. All participants were similar in age compared to the group of mothers (Table 1). DENV and chikungunya RNA were not detected in all tested patients, excluding coinfections.

### Detection of Dengue IgG and Zika Antibodies With an in-House ELISA

Serum samples were tested using ELISA IgG specific for ZIKV and DENV. Detection of dengue-specific IgG antibodies (Panbio, Australian) and Zika-specific IgG antibodies (Euroimmun, Germany) was performed according to the manufacturer's protocol. These ELISAs were standardized to be used as recommended after studies by the Brazilian Ministry of Health. However, there are concerns about the genuine potential for serological cross-reactivity as a confounding factor in ELISA screening.

### The Plaque Reduction Neutralization Test (PRNT)

The PRNT was performed for the laboratory confirmation of Zika cases. The ZIKV/H.sapiens/Brasil/ES2916/2015 strain identified in the State of Espírito Santo, Brazil was used. The cutoff value for PRNT positivity was defined as 90% (PRNT_90_). Samples with neutralizing antibodies for ZIKV were also submitted to PRNT_90_ for dengue virus serotype 1 (DENV-1 from West Pacific). Reference viruses were provided by Laboratório de Flavivírus (LABFLA) of Fiocruz from their arbovirus stocks. The PRNT_90_ was performed to determine the maximum plasma dilution (1:10–1:320) needed to reduce arbovirus plaque formation by 90% among Vero ATCC CCL-81 cells, following standard protocols (32, 33). All plasma was heat-inactivated (56°C, 30 min) before neutralization testing. Next, a final volume of each inactivated sample and virus mixture was transferred to a well-containing Vero cells and then initially screened at a dilution of 1:10 in 6-well-plates at 37°C for 60 min. Those that neutralized ZIKV by at least 90% were further tested at serial 2-fold dilutions to determine 90% endpoint titers. Plasma samples were considered to have DENV-neutralizing antibodies when a plasma dilution of at least ≥10 reduced no <90% of the formation of DENV viral plaques.

### PBMC Isolation

Briefly, peripheral blood mononuclear cells (PBMCs) and plasma were isolated by Ficoll-Paque PLUS density gradient centrifugation (GE Healthcare, United States) and frozen in fetal bovine serum (FBS, Gibco, Invitrogen Co, United States) containing 10% (vol/vol) dimethyl sulfoxide (DMSO) (Sigma-Aldrich, United States). Cells were thawed on the day of the experiment and were used directly for *in vitro* assay.

### ZIKV CD4 and CD8 MegaPools Description

ZIKV CD4 and CD8 megapool peptides (MP) have been designed and validated, as previously reported (34, 35). Briefly, a consensus sequence was generated by MAFFT alignment after querying the availability of NCBI polyprotein ZIKV sequences and BLAST to a corresponding ZIKV isolate being able to represent most of the viral sequence analyzed (ID: 64320) (36). Two different strategies have been then applied based on the ZIKV polyprotein sequence to predict CD4 and CD8 epitopes using the TepiTool (37) available in the immune epitope database analysis resource (IEDB-AR) (38). Specifically, to design the ZIKV CD4 MP, the “7-allele-method” (39) was applied with a cutoff of ≤ 20, while, for the ZIKV CD8 MP, the epitopes were predicted using the panel of 27 most frequent A and B alleles with custom selection to consider both 9-mers and 10-mers for the prediction (40) and consensus percentile rank cutoff ≤ 1.5. The resulting predicted epitopes have been separately clustered for CD4 and CD8 using an IEDB cluster 2.0 tool, applying the cluster-break method with a 70% cutoff for sequence identity (41). The corresponding peptides derived by the bioinformatic analyses were synthesized as a crude material (A&A, San Diego, CA), resuspended in DMSO, and pooled according to CD4 or CD8 MP composition followed by sequential lyophilization.

Thus, the MP approach was designed by taking into account the 27 most frequent HLA class I allelic variants worldwide, thus making it possible to capture reactivity independently from geographical location, as previously shown in the context of both ZIKV- and DENV-specific CD8+ T cell responses (25, 40, 42, 43). While we cannot rule out the possibility that the specific population considered might express an allelic variant that is infrequent in the worldwide population, the MPs are still designed to provide a worldwide population coverage of 90% or more.

### IFN-γ ELISpot Assay

Frozen collected PBMCs were assayed for IFN-γ cell responses, as previously described (44). Briefly, mouse anti-human IFN-γ antibody (clone 1-DK1; Mabtech) was added to 96-well-plates (Multiscreen HTS; Millipore) coated at 2.5 μg/ml, diluted in phosphate buffer saline (PBS), pH 7.2–7.4 (Sigma-Aldrich). PBMCs were then added in triplicate to wells (2 ×10^5^ cells/well) in the presence or not of ZIKV MPs at 1 μg/ml, followed by incubation at 37°C, 5% CO_2_ for 18–20 h. After washing with PBS–Tween 20, 1 μg/ml of IgG biotinylated anti-human IFN-γ (clone 7-B6-1; Mabtech) was added and incubated for 2 h at room temperature. After washing, streptavidin–alkaline phosphatase substrate (Mabtech) was prepared and added to the plate, for 1 h at room temperature. Plates were washed, and alkaline phosphatase substrate of 5-bromo-4-chloro-3-indolyl-phosphate/nitro blue tetrazolium chloride (BCIP-NBT) from KPL (Gaithersburg) was added after allowing spots to develop. The reaction was stopped by washing with tap water. Spots were counted using an automated ELISpot reader (ImmunoSpot1S6UV Ultra, Cleveland). The number of IFN-γ-producing cells was expressed as spot-forming cells (SFC) relative to 10^6^ PBMCs. Values were calculated by subtracting the number of spots detected in unstimulated control wells. Values were considered positive if they were equal or >20 spots and at least two times above the mean of unstimulated control wells. The stimulation with phytohemagglutinin (PHA, 5 μg/mL) was done for all individuals analyzed as a positive control of *in vitro* stimulation.

We have previously established an approach in which, prior to initiating the ELISpot assay, we recovered stimulated PBMCs for 20 h and stained with Abs listed in Supplementary Table 1 for extracellular staining used for flow cytometry experiments.

### Intracellular Cytokine Staining

Peripheral blood mononuclear cells (2 ×10^5^ cells/well) were cultured for 6 h with 1 μg/ml ZIKV MPs and brefeldin A (44). Subsequently, the stimulated PBMC were stained with the Abs used for flow cytometry experiments listed in Supplementary Table 1. The intracellular cytokine staining (ICS) was performed, permeabilized with saponin (0.05%), and stained with anti-IFNγ antibody. The stimulation with phorbol and ionomycin myristate acetate (PMA plus ionomycin) was performed on all individuals analyzed as a positive control of *in vitro* stimulation. The data were collected using BD FACSAria III flow cytometer and analyzed using FlowJo 10.5.2 software (Tree Star1, USA).

### Statistical Analysis

Comparisons between different groups were performed using either the non-parametric Mann–Whitney rank sum test or the parametric unpaired *t*-test (two-tailed analyses). When data followed a normal distribution, the paired Wilcoxon's test was used. Outcome variables were compared among the groups of study using the Kruskal–Wallis test followed by the Dunn's multiple comparisons test. Multiple comparison tests were used to compute *post-hoc* comparisons for all pairs of groups. The statistical significance of differences in frequency of “Responders” was performed using the Fisher's exact test. The planned statistical comparisons rely on the accurate classification of outcome and detection of arbovirus infections and CZS in infants. Data in all figure parts in which error bars are shown were presented as the median with interquartile range (25–75%). An analysis was performed with GraphPad PRISM (version 5) (GraphPad Software). The value of *p* < 0.05 was considered statistically significant.

## Results

### Detection of ZIKV and DENV-Specific IgG Antibodies by Commercial ELISA and of Neutralizing Antibodies to ZIKV and DENV

Zika virus (ZIKV)- and DENV-specific IgG antibody detection was initially determined using the commercial capture ELISA assay. Out of the 21 symptomatic mothers tested, who were qRT-PCR ZIKV+, 14 (66.7%) had both anti-ZIKV IgG and anti-DENV IgG. Similar percentages were observed between mothers who had asymptomatic children (8 out of 12; 66.7%) and those who had children with CZS (6 out of 9; 66.7%). Five out of 21 (23.8%) had anti-ZIKV IgG but not anti-DENV IgG, and similar percentages were also observed between those who had asymptomatic children (3 out of 12; 25%) and those who had children with CZS (2 out of 9; 22.2%). Only one mother who had a child with CZS (1 out of 21; 4.8%) had anti-DENV IgG but not anti-ZIKV IgG. Another mother who had a child with CZS (1 out of 21; 4.8%) had neither anti-DENV IgG nor anti-ZIKV IgG.

Regarding the 18 children with a history of intrauterine exposure to ZIKV, it was not possible to perform the assay on one child with CZS because the sample volume was insufficient. Most of the children, i.e., 10 out of 17 (58.8%), had neither anti-DENV IgG nor anti-ZIKV IgG, among whom seven were asymptomatic (7 out 9; 77.8%) and three had CZS (3 out of 8; 37.5%). Five out of 17 (29.4%) had anti-DENV IgG but not anti-ZIKV IgG, among whom one was asymptomatic (1 out 9; 11.1%) and four had CZS (4 out of 8; 50%). Two out of 17 (11.8%) had both anti-ZIKV IgG and anti-DENV IgG: one was asymptomatic (1 out of 9) and another had CZS (1 out 8). None of them had anti-ZIKV IgG but not anti-ZIKV IgG.

To confirm previous ZIKV and DENV exposure, plasma samples were tested using PRNT_90_ for the detection of ZIKV and DENV-1-neutralizing antibodies. We could not perform PRNT for all four DENV serotypes since the plasma volume was insufficient for this. Thus, we chose to evaluate DENV-1 since this had the highest prevalence in Rio de Janeiro in the period in which the samples were collected (2015–2016) (45). Out of 19 mothers who had anti-ZIKV IgG, 18 (94.7%) presented with ZIKV-neutralizing antibodies with PRNT_90_ titer > 320, thus confirming the exposure to ZIKV. Interestingly, out of 14 mothers who had both anti-ZIKV IgG and anti-DENV IgG that were detectable, 10 had a PRNT_90_ titer for ZIKV and DENV, thus indicating exposure to both viruses, while 3 presented a PRNT_90_ titer for ZIKV and 1 did not have any detectable PRNT_90_ titer. Regarding the children, it was not possible to perform PRNT on three of them because the sample volume was insufficient. Thus, two out of 15 children had both anti-ZIKV IgG and anti-DENV IgG that were detectable, and 1 asymptomatic child presented both ZIKV and DENV-neutralizing antibodies.

The frequency of T cell subpopulations was assessed by flow cytometry assay. Data on the percentage of CD4 and CD8 T cells and the ratio between the two subpopulations showed no statistically significant difference between the two donor cohorts (Table 1).

### Detection of IFN-γ-Producing Cells in Response to ZIKV Peptides in Women and Mothers With a History of ZIKV Infection, but Not in Children With a History of Intrauterine Exposure to ZIKV

Next, we focused on the analysis of T cell responses in donors with a history of ZIKV infection. *Ex vivo* T cell responses to CD4 and CD8 ZIKV MPs (CD4 ZIKV MP and CD8 ZIKV MP) employing the ELISpot assay were measured to quantify the number of IFN-γ secreting cells. The general characteristics of these donors' cohorts are summarized in Table 1.

**Figure 2:**
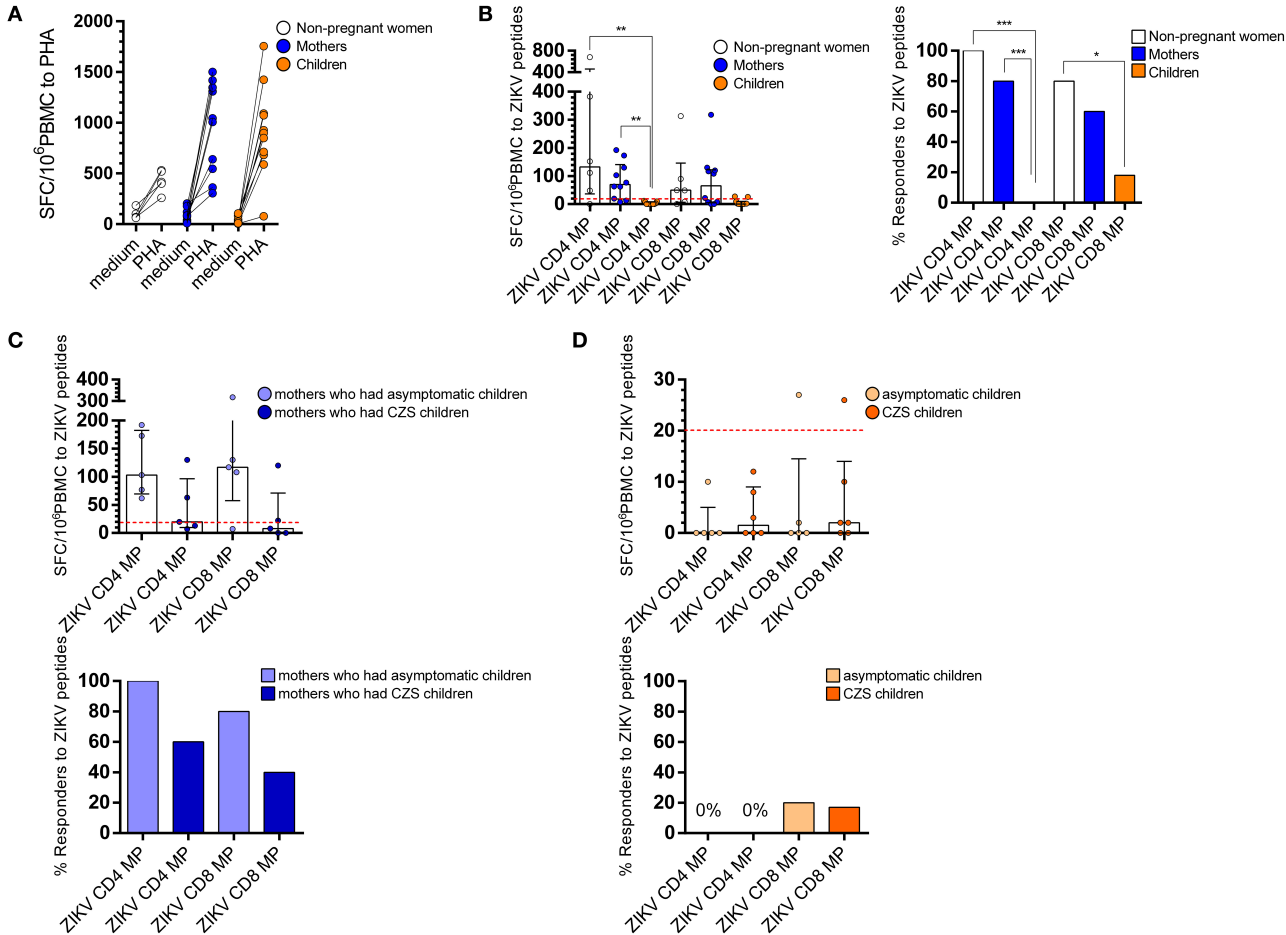
*Ex vivo* reactivity to CD4 and CD8 ZIKV MPs in donors with a history of ZIKV infection. T cell response to by ELISpot *ex vivo* experiments are shown for women (from non-pregnant women infected with ZIKV, white, *n* = 5), mothers (from pregnant mothers infected with ZIKV, blue, *n* = 10), and children (from children born to mothers infected with ZIKV during pregnancy, orange, *n* = 11) with a history of ZIKV infection. In **(A)**, T cell response to PHA stimulation and, in **(B)**, the magnitude of CD4 and CD8 T cell reactivity to ZIKV MPs are expressed as medians and interquartile range (25th and 75th percentiles). Donors were considered “Responders” if the net SFC/106 PBMC after ZIKV MP was ≤ 20 after 20 h of *in vitro* stimulation. **(C)** CD4 and CD8 T cell reactivity to a ZIKV MP are shown among mothers who had asymptomatic children (light blue) compared with those who had CZS children (dark blue). **(D)** CD4 and CD8 T cell reactivity to ZIKV MPs are shown among children grouped in asymptomatic children (light orange) compared with CZS children (dark orange). Responses were expressed as the number of IFN-g-secreting cells per 106 PBMC. Statistical differences were tested using the one-way analysis of variance (ANOVA) and the Kruskal-Wallis test followed by the Dunn's multiple comparisons test. Bars represent median with interquartile range. Statistical significance for differences in the frequency of “Responders” was performed using Fisher's exact test. ^*^ *p* < 0.05, ^**^p < 0.001, ^***^p < 0.001. ZIKV, Zika virus; MP, megapool peptide; CZS, congenital Zika syndrome; PBMC, peripheral blood mononuclear cell.

To ensure the comparable quality of samples, we first compared responses induced by the positive control stimulus PHA. As expected, T cells from women (non-pregnant women infected with ZIKV, see Figure 1 in Materials and Methods) and mothers (pregnant mothers infected with ZIKV, see Figure 1 in Materials and Methods) with a history of ZIKV infection responded to PHA with values above 250 SFC per 10^6^ PBMC, as well as most of the children with a history of intrauterine exposure to ZIKV (from children born to mothers infected with ZIKV during pregnancy, see Figure 1 in Materials and Methods). One of the samples collected from children responded to PHA with just over 70 SFC per 10^6^ PBMC, which resulted in 15 times greater stimulation with PHA compared to the medium; therefore, we decided to maintain this child from the analysis (Figure 2A).

In the ELISpot assay, we were able to successfully detect T cell responses using CD4 ZIKV MP and CD8 ZIKV MP. The T cells of all non-pregnant women responded to ZIKV CD4 MP and four out of the five responded to CD8 MP. The frequency of responders was slightly lower in mothers, so the CD4 T cells of 8 out of 10 mothers responded to ZIKV CD4 MP and 6 out of 10 responded to ZIKV CD8 MP. These data demonstrated an important reactivity of donors exposed to ZIKV, both in frequency and magnitude of CD4 and CD8 T cell responses to ZIKV peptides. However, children's T cell responses to ZIKV MP were uncommon as no child responded to CD4 ZIKV MP and only 2 out of 11 responded to CD8 ZIKV MP (Figure 2B).

The T cells of all mothers who had asymptomatic children responded to CD4 ZIKV MP and 4 out of the 5 responded to CD8 ZIKV MP. Among those who had children with CZS, the T cells of 3 out of 5 responded to CD4 ZIKV MP and 2 out of 5 to CD8 ZIKV MP. No significant difference was found between the two groups of mothers regarding the magnitude or frequency of responses to the ZIKV MP (Figure 2C).

Regarding children, regardless of the clinical outcome, T cells did not respond to CD4 ZIKV MP, and only 1 out of 5 asymptomatic and 1 out of 6 with CZS responded to the CD8 ZIKV MP (Figure 2D).

### Higher CD4 T Cell Degranulation Ability Compared to CD8 T Cell Degranulation in Donors With a History of ZIKV Infection

**Figure 3:**
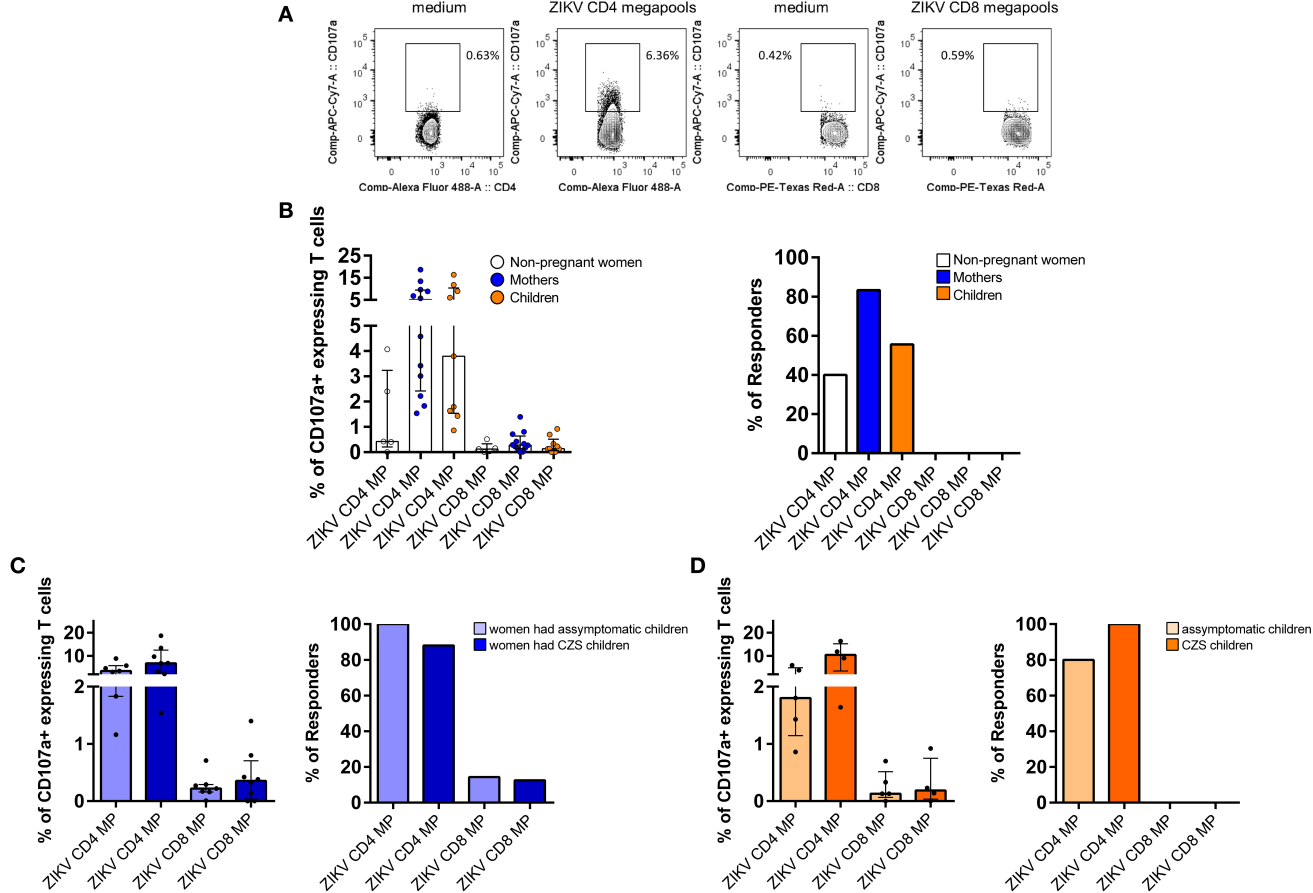
Degranulation activity of T cells from donors with a history of ZIKV infection after ZIKV MP *in vitro* stimulation. **(A)** Gating strategies to the flow cytometry profiles of the degranulation activity of CD4 and CD8 T cells measured by CD107a mobilization after stimulation with ZIKV MPs are shown in a representative of one of the women (from non-pregnant women infected with ZIKV, white, *n*= 5), mothers (from pregnant mothers infected with ZIKV, blue, *n*= 12), and children (from children born to mothers infected with ZIKV during pregnancy, orange, *n*= 9) with a history of ZIKV infection. **(B)** Cumulative data (medians and interquartile range) of the proportion of CD107a+ cells among CD4 and CD8 T cells after ZIKV MP stimulation. Regarding the criteria of positivity of the assay or “Responder,” the background in the unstimulated controls was subtracted to ZIKV peptide conditions, and the responder was considered as those who had two times more CD107a-positive cells after subtraction of the background. **(C)** CD4 and CD8 T cell reactivity to ZIKV MPs are shown among mothers who had asymptomatic children (light blue) compared with those who had CZS children (dark blue). **(D)** CD4 and CD8 T cell reactivity to ZIKV MPs are shown among asymptomatic children (light orange) compared with CZS children (dark orange). The magnitude of responses is expressed in medians and interquartile range, and statistical analyses were performed with Mann-Whitney U-test. No statistical difference was found. ZIKV, Zika virus; MP, megapool peptide; CZS, congenital Zika syndrome.

Cytotoxic activity is one of the key components of the virus-specific protective immunity (25). In this study, we measured the degranulation capacity of T cells by staining of the expression of the CD107a molecule (Figure 3A). Our results were very surprising since we detected an increase in the magnitude of the CD107a-expressing CD4 T cells after stimulation with CD4 ZIKV MP. This response was more intense in mothers and children and less in women. Thus, regarding the response of CD4 T cells to CD4 ZIKV MP, 10 out of 12 mothers, 5 out of 9 children and 2 out of 5 women were responsive to CD4 ZIKV MP with an increase in the frequency and magnitude of CD107a-expressing CD4 T cells. In contrast, no donor responded to CD8 ZIKV MP by inducing the CD107a-expressing CD8 T cell responses (Figure 3B).

Further analysis revealed that the response of CD4 T cells to CD4 ZIKV MP was similar among mothers who had asymptomatic or SCZ children (Figure 3C). Among children, this response was also independent of whether they had neurological impairment associated with ZIKV (Figure 3D).

### Higher IFN-γ Producing ZIKV CD4 T Cell Responses Persist in Donors With a History of ZIKV Infection by ICS Assay

**Figure 4:**
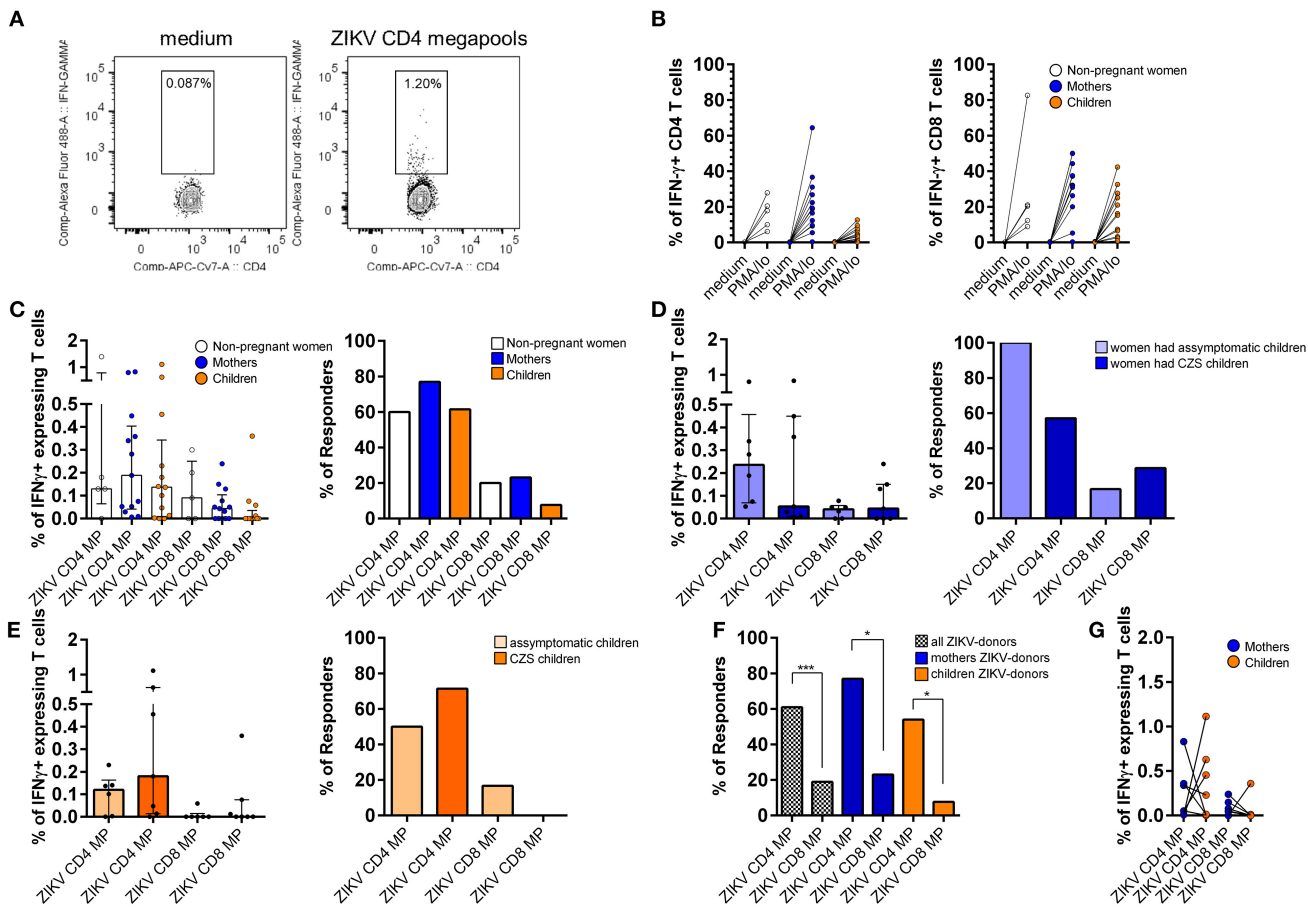
*Ex vivo* reactivity with IFN-γ production of donors with a history with ZIKV infection to CD4 and CD8 ZIKV MPs. CD4 and CD8 ZIKV-restricted responses in women (from non-pregnant women infected with ZIKV, white, *n* = 5), mothers (from pregnant mothers infected with ZIKV, blue, *n* = 13), and children (from children born to mothers infected with ZIKV during pregnancy, orange, *n* = 13) with a history of ZIKV infection after 6-h of *in vitro* stimulation. **(A)** Gating strategy to the flow cytometry plots identifies IFN-g-producing ZIKV-specific T cells. **(B)** Each data point represents the response of a single donor tested with the PMA + io and **(C-E)** two different sets of two protein pools, ZIKV CD4 MP and ZIKV CD8 MP. **(C)** Magnitude of CD4 and CD8 responses and frequency of responders (right) were performed from women, mothers, and children donors to the ZIKV CD4 or CD8 MPs. **(D,E)** Each group is further divided into asymptomatic or CZS. **(D)** Magnitude of CD4 and CD8 responses (left) and frequency of responders (right) were performed from mothers who had asymptomatic children (light blue, *n* = 6) and those who had children with CZS (dark blue, *n*= 7) to the ZIKV CD4 or CD8 peptides. **(E)** Magnitude of CD4 and CD8 responses (left) and frequency of responders (right) were performed from asymptomatic children (light orange, *n*= 6) and CZS children (dark orange, *n* = 7) to the ZIKV CD4 or CD8 MPs. **(F)** Frequency of responders were performed from all (dashed white, *n* = 31), mothers (blue, *n* = 13), and children (orange, *n* = 13) donors to the ZIKV CD4 or CD8 MPs. **(G)** The frequencies of ZIKV-specific IFN-γ-positive CD4 and CD8 cells were compared between the mother-child pairs (tested pairs *n* = 7). The p-values were calculated using the paired t-test. The magnitude of responses is expressed medians and interquartile range, and statistical analyses were performed with the Mann-Whitney U-test. Statistical significance for differences in frequency of responders was performed using a Fisher test. Asterisks indicate significant differences (^*^p < 0.05, ^***^p < 0.001). ZIKV, Zika virus; MP, megapool peptide; CZS, congenital Zika syndrome; INF, interferon.

By using a ZIKV MP stimulation and ICS assays (Figure 4A), we were able to determine the responding T cell subsets as well as the memory subset. Donors cells with a history of ZIKV infection were stimulated with polyclonal PMA plus ionomycin, confirming the viability of all samples (Figure 4B).

In a further series of experiments, CD4 T cells from all donors responded to the CD4 ZIKV MP in terms of frequency and magnitude of responses.

CD4 T cells responded to the CD4 ZIKV MP with IFN-γ production in most donors exposed to ZIKV; 3 out of 5 women, 10 out of 13 mothers, and 8 out of 13 children. Regarding the responses of CD8 T cells after stimulation with CD8 ZIKV MP, we observed a lower frequency of responders in all groups: only one out of 5 women, 3 out of 13 mothers, and only 1 out of 13 children (Figure 4C). Thus, all donors showed appreciable reactivity, both in terms of increased frequency and magnitude of IFN-γ-producing CD4 responses but a lower response for CD8 responses.

We went to see if there is a difference between the groups of mothers and children. The CD4 T cells of all mothers who had asymptomatic children responded to CD4 ZIKV MP with IFN-γ production, while 4 out of 7 mothers who had CZS children responded, but this difference was not statistical. Regarding CD8 T cell responses, CD8 ZIKV MP responses were remarkably close (Figure 4D). Among children, T cell responses to ZIKV MP were quite similar, therefore, regardless of whether they had any neurological impairment associated with ZIKV (Figure 4E).

Regarding the IFN-γ producing CD4 and CD8 T cells, we observed that there is a greater degree of reactivity of CD4 T cells after CD4 ZIKV MP compared to CD8 T cells after CD8 ZIKV MP in all studied groups (Figure 4F and Supplementary Figure 1). We performed an analysis of the mother and child pairs to assess whether the responses agreed (pairs tested *n* = 7). Only two of the seven mother and child pairs responded to CD4 ZIKV MP. For the response of CD8 T cells to CD8 ZIKV MP, in 4 pairs, mothers and children did not respond to CD8 ZIKV MP and, in three cases, mothers responded, but children did not (Figure 4G).

When the ELISpot and ICS data for all mothers, relating to CD4 ZIKV MP or CD8 ZIKV MP, were combined, we found a significant positive correlation between ELISpot and ICS (*p* = 0.002 for CD4 ZIKV MP and *p* = 0.008 for CD8 ZIKV MP; the Wilcoxon signed-rank test). Thus, in a broad and collective sense, ICS does not fundamentally conflict with ELISpot in the group of mothers. A similar analysis was performed for children, but no significant correlation between ELISpot and ICS was found for either CD4 ZIKV MP or CD8 ZIKV MP (the Wilcoxon signed-rank test). In the ELISpot 20-h cultures, we evaluated susceptibility to death of PBMCs. In particular, the children's PBMCs had a viability ≥100%. We speculate that the kinetics of cytokine production may have more influence on children's PBMCs than on those of their mothers. Other kinetics should therefore be evaluated in future studies.

Additionally, we have measured IL-8 levels by ELISA in PBMC supernatants from ZIKV donors recovered from the 20-h cultures for the ELISPOT assay. No statistical difference was found between the groups when comparing the different conditions (data not shown).

### The Memory CD4 T Cells Re-Expressing CD45RA (Temra) in Donors With a History of ZIKV Infection Is Enriched for ZIKA-Specific T Cells

**Figure 5:**
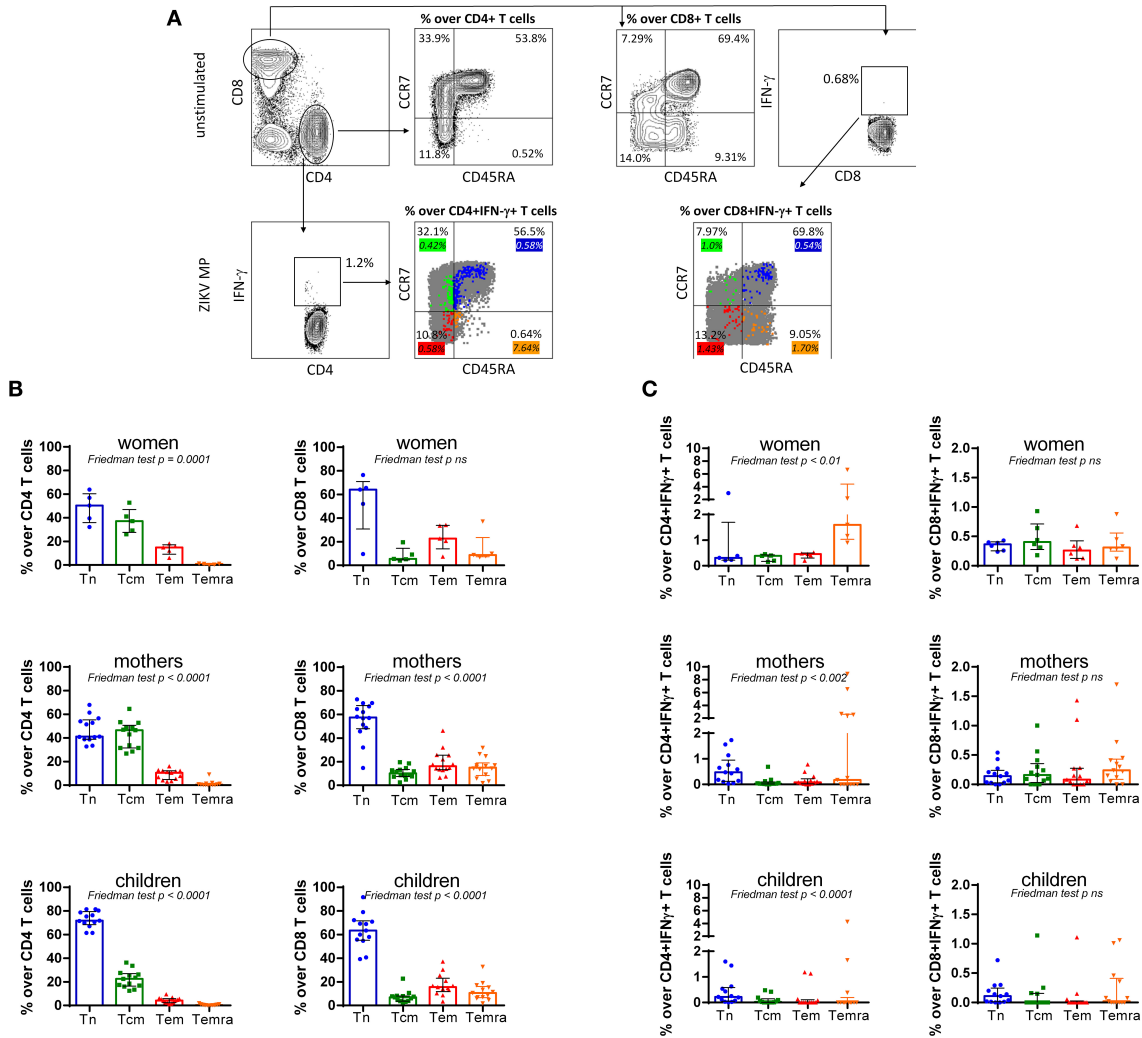
Subpopulations of specific memory from ZIKV donors with a history of ZIKV infection. (A) Gating strategy to identify the CD4 T cell and CD8 T cell subsets, and of the T cell memory subpopulations. Lymphocytes were identified and electronically gated on orthogonal light scatter signals and CD3 immunopositivity. Then, CD3+CD4+T cells and CD3+CD8+T cells were identified. Gating on CD3+CD4+T cells or CD3+CD8+T cells, Tn and Tcm cells were identified as CD45RA negative/positive and CCR7 negative/positive. In unstimulated cells, the percentage of unspecific CD4 and CD8 T cell subsets [Tn: CD45RA+CCR7+; Tcm: CD45RA−CCR7+; effector memory (Tem): CD45RA−CCR7− and effector memory re-expressing CD45RA T cells (Temra): CD45RA+CCR7−] was gated in a representative subject with a history of ZIKV. The percentage values are represented in the graphs. To determine the memory T cell responses to ZIKV MP, the percentage of CD4 and CD8 T cell subsets were demonstrated. The color values represent the frequency of the responding IFN-γ-secreting Tn out of the total of Tn cells, responding IFN-g-secreting Tcm out of the total of Tcm cells, responding IFN-γ-secreting Tem out of the total of Tem cells, and responding IFN-γ-secreting Temra out of the total of Temra cells. The color percentage values are represented in the graphs. **(B)** Percentage of unspecific CD4 and CD8 T cell subsets (Tn, Tcm, Tem, and Temra) gated in unstimulated cells from women (from non-pregnant women infected with ZIKV), mothers (from pregnant mothers infected with ZIKV), and children (from children born to mothers infected with ZIKV during pregnancy) with a history of ZIKV. **(C)** The phenotypic analysis of the responding IFN-γ-secreting T cells for ZIKV epitopes out of a total of Tn or Tcm cell subsets in women, mothers, and children. Data are expressed as median ± the interquartile range for each cohort (*n*= 5-13 in both panels). Differences between T cell subsets among cohorts were analyzed using the Friedman test. Each data point represents a single donor determination. ZIKV, Zika virus; MP, megapool peptide; CZS, congenital Zika syndrome; INF, interferon; Tn, naïve T cell; Tcm, central memory T cell; Tem, effector memory T cell; Temra, effector memory re-expressing CD45RA T cell.

We initially analyzed the expression of CD45RA and CCR7 on the total CD4 and CD8 population (Figure 5A). In women, the majority of the unspecific CD4 T cells displayed a frequency of naïve (Tn, CD45RA+CCR7+; 50%) phenotype followed by the central memory (Tcm, CD45RA-CCR7+; 37%) phenotype, effector memory (Tem, CD45RA–CCR7–; 15%) phenotype, and finally by effector memory re-expressing CD45RA (Temra, CD45RA+CCR7–; 0.9%) phenotype. In mothers, it was observed that a similar frequency of the unspecific CD4 T cells displayed a Tcm (47%) phenotype and a Tn (41%) phenotype, and then an Tem (11%) phenotype, followed by a Temra (0.5%). As expected, in children, the most CD4 T cells exhibited a Tn (72%) phenotype, followed by a Tcm (23%) phenotype, an Tem (4%) phenotype, and an Temra (0.4%) phenotype (Figure 5B).

For the unspecific CD8 T cells, women, mothers, and children had a comparable frequency of all subsets of T cells, exhibiting predominantly a Tn cell (64% in women, 58% in mothers, and 63% in children), followed by a Tem phenotype (23% in women and 16% in both mothers and children donors), a Temra (9, 15, and 11%, respectively), and lastly a Tcm cell (6, 10, and 7%, respectively) (Figure 5B). Thus, the majority of unspecific CD4 and CD8 T cells are in the Tn population.

To further determine the memory T cell responses to ZIKV MP, we compared it to responding IFN-γ-secreting Tn out of the total of Tn cells, responding IFN-γ-secreting Tcm out of the total of Tcm cells, responding IFN-γ-secreting Tem out of the total of Tem cells, and finally responding IFN-γ-secreting Temra out of the total of Temra cells (Figure 5A).

Further analysis demonstrated that phenotypic analysis of the total of Temra subsets is enriched for responding IFN-γ-secreting CD4 T cells for ZIKV epitopes in women, mothers, and children (Figure 5C). In terms of the IFN-γ+-responding CD8 T cells for ZIKV epitopes, no difference was seen in the frequency of Tn or Tcm phenotype in donors with a history of ZIKV infection (Figure 5C).

## Discussion

In the present study, we aimed to evaluate the T cell response in donors with a history of ZIKV infection during pregnancy. Regarding the few current studies that raise the same questions, our group has progressed when compared to non-pregnant women, pregnant mothers, and both asymptomatic and CZS children born to mothers infected with ZIKV during pregnancy.

It is essential to understand the repertoires of the ZIKV effector T cell response to the point of extrapolating if the development of a non-protective immunity in mothers is related to the affected children. In addition, in pregnancy and congenital infection scenarios, it is not known if there is a development of long-lasting T cell immunity to ZIKV. To address these issues, the ZIKV T cell immunity in a cohort of mothers infected with ZIKV during pregnancy and children affected (or not) by neurological complications was assessed 2–3 years after the initial infection.

If previous DENV cross-reactive immunity contributes or not to protection against ZIKV infection during pregnancy, it is still unclear. During the Brazilian ZIKV epidemic (2016–2017), 94 pregnant women who gave birth to infants with or without microcephaly were evaluated. Based on previous studies, DENV immunity is not a prerequisite for ZIKV entry into the fetal compartment (46). Halai et al. supported Araújo's findings (47). In contrast, ZIKV-infected human placental tissues showed increased replication in the presence of DENV antibodies, dependent on FcγR engagement (48). In mouse models, the presence of DENV-specific antibodies in ZIKV-infected pregnant mice significantly increased fetal resorption in immune-compromised mice (48) and also displayed CZS, including microcephaly, in immune-competent mice (49, 50).

Thus, conflicting datasets regarding the impact of ADE (antibody-dependent enhancement) on congenital malformations in babies have emerged from different studies, including mouse and non-human primates' models and human donors. Based on Zimmerman's review, additional prospective epidemiologic studies, *ex vivo* studies, and animal model experimentation are needed to fully dissect the complex risk factors and pathways through which ZIKV can seed and infect the fetal compartment (51).

Even so, this can be framed if the mothers' ZIKV T cell effector immune response repertoires are “weak” to the point of not giving protective immunity to the affected children. So far, data addressing this issue is only available in the murine model (52). The authors evaluated adapted mouse models established for ZIKV infection during pregnancy. Interestingly, they observed a reduction in a load of ZIKV in maternal and fetal tissues and an increase in fetal viability and growth in immune mice to DENV compared to non-immune mice. The depletion or genetic deficiency of CD8 T cells nullified this effect, even under ADE conditions (52). A single study on pregnancy and immunity to ZIKV in human patients was recently developed by Reynolds et al. The authors showed that almost all mothers with a history of ZIKV infection have serological evidence of immunity to dengue, suggesting that their children who had microcephaly were born to mothers' immune to DENV. In addition, they showed that different viral products can induce a multifunctional response to E and NS1 antigens and also immunoregulatory responses to NS5 and C ZIKV proteins, which could impact immunopathogenesis (53). From our dataset, it is not yet clear if children who had microcephaly were born of mothers with “an altered” T cell responses. It would be necessary to have acute samples of pregnant mothers to answer this question with greater propriety.

Outside the context of donors infected during pregnancy or by congenital infection, several studies have been evaluating the T cell responses to ZIKV proteins in human donors immune or not to DENV. It is known that ZIKV-reactive T cells in the acute phase of infection are detected earlier and in greater magnitude in DENV-immune patients. Conversely, the pattern of CD4 responses to ZIKV-restricted class II peptides was remarkably similar in acute and convalescent phases, while a lower frequency and magnitude of responses were observed with the CD8 counterpart (44). In another study, Grifoni's data indicated an immunological signature for CD8 T cell responses reproducible and temporally stable after Ag-specific stimulation even 1–2 years after ZIKV infection (25). Moreover, circulating tetramer-positive ZIKV-specific CD8 T cells peaked at early convalescence day post-infection with elevated levels persisting for months from a volunteer woman (54). On the other hand, whereas C-, prM-, E-, and NS5-specific cytokine-expressing CD4 T cells were readily detected in all patients tested, absent or low detection of functional CD8 effector T cells against peptides spanning ZIKV C, prM, E, and NS5 was identified (55). We agree with Lai's study using peptides that span all 10 ZIKV proteins. Our data indicate that after *in vitro* stimulation with the ZIKV MP, CD4 T cells maintain their degranulation and IFN-γ production capacity, which was not observed by CD8 T cells. One possibility is that the cytotoxic and pro-inflammatory activities of CD8 T cells are high at an early stage of ZIKV infection but do not persist in the disease resolution phase when the virus has apparently been eliminated. Our experimental design was based on well-established protocols, with stimulation with ZIKV or DENV MP of cells cultured for 6 h to perform a flow cytometry assay or for 20 h for the ELISpot assay (25, 34, 42, 44, 56). Additionally, studies have already been carried out on *in vitro* expansion of peptide-specific T cells for 14 days (34, 56, 57). Thus, our future proposal is to perform *in vitro* expansion of peptide-specific T cells in the kinetics of up to 14 days, in order to confirm our observations on the role of CD8 T cells.

The protective role of CD8 T cells has been demonstrated in several studies using mouse models. Huang's study demonstrated that an adoptive transfer of ZIKV-immune CD8 T cells can protect against ZIKV infection (58). Indeed, polyfunctional, cytotoxic CD8 T cells are activated (59), reducing ZIKV burden, and, in the same sense, CD8 depletion or genetic absence resulted in greater ZIKV infection and mortality in mice (60). Moreover, ZIKV-specific and ZIKV/DENV cross-reactive CD8 T cells in DENV-immune mice expanded post-ZIKV challenge and reduced infectious ZIKV levels, and CD8 T cell depletion confirmed this protection (61). Nevertheless, within a susceptible mouse model, CD8 T cells appear as an important mediator of ZIKV neuropathogenesis (62, 63). It is currently impossible to determine what the impact of the short duration of ZIKV-specific CD8 T cells will be for donors with a history of ZIKV infection. Unfortunately, only with the emergence of new ZIKV epidemics, this issue can be assessed.

In relation to ZIKV-specific CD4 T cell responses in Zika patients, Koblischke et al. showed a strong impact of structural ZIKV protein on immunodominant CD4 T cell responses (64). Interestingly, most of the CD4 Temra cells are DENV-specific in donors with previous DENV infection (43). Patil et al. revealed in patients with dengue that these CD4 T cells in the Temra subsets are highly enriched with CD4 cytotoxic T lymphocytes expressing several genes linked to cytotoxic and costimulatory function, compared with CD4 T cells in the Tcm and Tem subsets (65). Tian et al. revealed that the CD4 Temra cells can be subdivided into two major subsets between DENV-immune individuals based on the expression of the adhesion G protein-coupled receptor GPR56. While GPR56+ Temra cells display a transcriptional and proteomic program with cytotoxic features, GPR56+ Temra cells have higher levels of clonal expansion and contain the majority of virus-specific Temra cells (66). Overall, like DENV infection, our data indicate that the Temra CD4 T cell subset is enriched for responding IFN-γ-secreting CD4 T cells for ZIKV epitopes in donors with a history of ZIKV infection. Future efforts will be underway in our laboratory to assess whether the heterogeneity of CD4 Temra T cells could provide insights to differentiate the outcome of the child's congenital infection in T cell responses against ZIKV.

Some limitations should be considered when interpreting our findings. First, to understand the evolution of the acquired ZIKV immunity, the best would be a “longitudinal study” in which donors were monitored in the acute and convalescent phases and after complete recovery from the disease. Second, the diagnosis of ZIKV through qRT-PCR was not made in newborns, so we cannot affirm that they were vertically infected, especially in asymptomatic cases. Recently, Ribeiro et al. published data from analyses on the positivity of PRNT and serological tests among a total of 88 children in northeastern Brazil who presented clinical cases of CZS between 2016 and 2018. Six (6.8%) were positive for IgG anti-ZIKV, and 2 (2.3%) of them were ZIKV PRNT_90_-positive (67). Although our initial goal was not to make correlations like those made by Ribeiro et al., our data are very close. In our study, only 2 out of 17 children (11.8%) had anti-ZIKV IgG (but also anti-DENV IgG), and 2 out of 15 (13.3%) were ZIKV and DENV PRNT_90_-positive. This frequency of positivity was much lower than we expected, as were the data of Ribeiro et al. Like in their study, our data cannot be attributed to problems in the execution of PRNT_90_, since the protocol established by Baer and Kehn-Hall (36) was strictly followed. The ZIKV/H.sapiens/Brasil/ES2916/2015 strain was identified in the State of Espírito Santo at the time of the CZS epidemic in Brazil (68). In a case report, it was shown that a child was positive for ZIKV IgG at birth and at the age of 12 months but was negative at 21 and 24 months of age. The child's mother was ZIKV RT-PCR, IgM, IgG, and PRNT-positive during the pregnancy. Three hypotheses were raised by the authors of that study: (a) low and transient ZIKV viremia could lead to the lack of detection of the antigen and absence of a strong immune response in the child; (b) specific and direct tropism of ZIKV to the central nervous system could cause ZIKV to become a silent virus that would escape the host's response; and (c) ZIKV could prevent the triggering of a strong innate immune response because active viral replication might have ceased during intrauterine life or shortly thereafter (69). Moreover, methodologically, the use of the recombinant NS1 antigen as a target in the commercial kit may explain the low anti-ZIKV IgG seropositivity in newborns (70).

Third, it was not possible to carry out many analyzes of paired mothers and children, mainly due to the clinical complications of the children during the study, which made it difficult to define a close association between the immunity of mothers and the clinical outcome of their children. Fourth, a caveat to this study is that the T cell responses detected after ZIKV MP restimulations may actually cover specific ZIKV responses and responses from cross-reactions due to previous flavivirus infections, particularly DENV.

Given the similarity between DENV and ZIKV and their common regions of endemicity, questions have arisen about whether immunity to one virus can provide cross-protection against another. Grifoni et al. published data that demonstrated that the degree of MP sequence homology for different flaviviruses directly influenced cross-reactivity in terms of CD4 and CD8 T cell responses. It is important to note that the CD8 MP of ZIKV used in our study was provided by Dr. Sette's team and, therefore, is the same as used by Grifoni et al. According to them, 33% of the DENV and ZIKV CD8 MP have homology ≥70% of sequence similarity with the consensus sequence, which in fact means that one-third of the peptides present in the MPs that we tested can cross-react with DENV (34). Here, our data showed that more than 70% of mothers who had both anti-ZIKV IgG and anti-DENV IgG had a PRNT_90_ test positive for ZIKV and DENV. Among the children, 50% of those who had both anti-ZIKV IgG and anti-DENV IgG presented ZIKV and DENV-neutralizing antibodies. Several human studies have investigated cross-reactivity in T cell responses, targeting a variety of viral proteins (71). Grifoni et al. found that prior DENV exposure did not have any impact on the CD4 T cell response to ZIKV infection: patients in the acute phase of ZIKV infection had more IFN-γ+ CD8 T cells after restimulation with ZIKV-derived peptides (44). However, prior DENV immunity did not have any impact on either the transcriptional profile of the CD8 T cells or the capacity of CD4 or CD8 T cells to produce IFN-γ at later stages of infection (25). Therefore, we have no way of knowing with certainty whether the T cell response that we are evaluating is directed only to primary ZIKV infection and not to DENV. We believe that the most important thing is that, despite the high degree of cross-reactivity between T cell responses to DENV and ZIKV, no indication has yet been provided that this may have a negative impact on disease outcomes.

Our data did not show any notable differences between children with CZS and asymptomatic children or between mothers who gave birth to children with CZS and those who gave birth to asymptomatic children, 2–3 years after primary infection. Recently, Macedo-da-Silva et al. used the same cohort of children as in our study, also in the recovery phase. They demonstrated that asymptomatic children presented changes in proteins that participated in processes relating to neuronal death and cerebrovascular abnormalities, low regulation of proteins relating to vision, and increased activity of metalloproteinases, namely MMP-2 and MMP-9, associated with neuronal death. Interestingly, these changes are not common among control children, but they are also characteristic of children with CZS (72). Together with Macedo-da-Silva et al. we think that, perhaps, marked differences between the groups occurred in the acute phase of Zika but were not so evident in the recovery phase.

In summary, we studied donors with a history of ZIKV infection that occurred in the 2016–2017 outbreak in Brazil, including non-pregnant women, mothers infected with ZIKV during pregnancy, and children with a history of intrauterine exposure. In general, donors demonstrated long-term immunity from CD4 T cells to ZIKV CD4 MP, but the same was not observed in CD8 T cells with ZIKV CD8 MP. It is possible that the cytotoxic and pro-inflammatory activities of CD8 T cells are markedly exhibited in the early stages of the disease but less detected in the resolution phase. The responses of mothers' T cells and children's T cells to ZIKV MPs do not appear to be related to the clinical outcome. These data still need to be investigated, including the evaluation of CD8 T cell response to other ZIKV peptides.

## Study Approval

These patients were recruited from the Exanthematic Diseases Unit at the Hospital Universitário Antonio Pedro of the Universidade Federal Fluminense, Niteroi city, RJ, Brazil. All activities were performed after obtaining the informed consent of mothers and parents of children. This approved study is titled “Clinical follow-up of pregnant women with rash and their children: prospective study cohort” with approval number 56913416.9.0000.5243.

## Data Availability Statement

The raw data supporting the conclusions of this article will be made available by the authors, without undue reservation.

## Ethics Statement

The studies involving human participants were reviewed and approved by these patients were recruited from the Exanthematic Diseases Unit at the HUAP/UFF, Niteroi city, RJ, Brazil. All activities were performed after obtaining the informed consent of mothers and parents of children. This approved study is titled Clinical follow-up of pregnant women with rash and their children: prospective study cohort with approval number 56913416.9.0000.5243. Written informed consent to participate in this study was provided by the participants' legal guardian/next of kin.

## Author Contributions

JB-C, IAP and, DF-M developed and performed experiments, analyzed, and interpreted data. HGD, AP-C, and CF-S clinically characterized patients for the study including the PRNT90 trials for ZIKV and DENV-1 and additional cytokines ELISA. MG made the sample acquisitions on the flow cytometer. FRC, AAS, RAOV, and CAAC recruited the clinical cohort for the study. FRC, MRQL, AAS, SMBC, and CAAC clinically characterized patients for the study including DENV IgG and ZIKV IgG ELISA. SAO, ELA, and CAAC helped prepare the manuscript. AG, AS, and DW designed and validated the ZIKV megapools. LMO-P and JB-C conceived and designed the study, interpreted the data, and wrote the manuscript. CAAC and LMO-P supervised the research. All the authors discussed the results and commented on the manuscript.

## Conflict of Interest

The authors declare that the research was conducted in the absence of any commercial or financial relationships that could be construed as a potential conflict of interest.
