# Supplementary figures and images for: Differential Longevity of Memory CD4 and CD8 T Cells in a Cohort of the Mothers With a History of ZIKV Infection and Their Children
Source: Front Immunol. 2021 Feb 12;12:610456. doi: 10.3389/fimmu.2021.610456 (PMC7928292; doi:10.3389/fimmu.2021.610456)

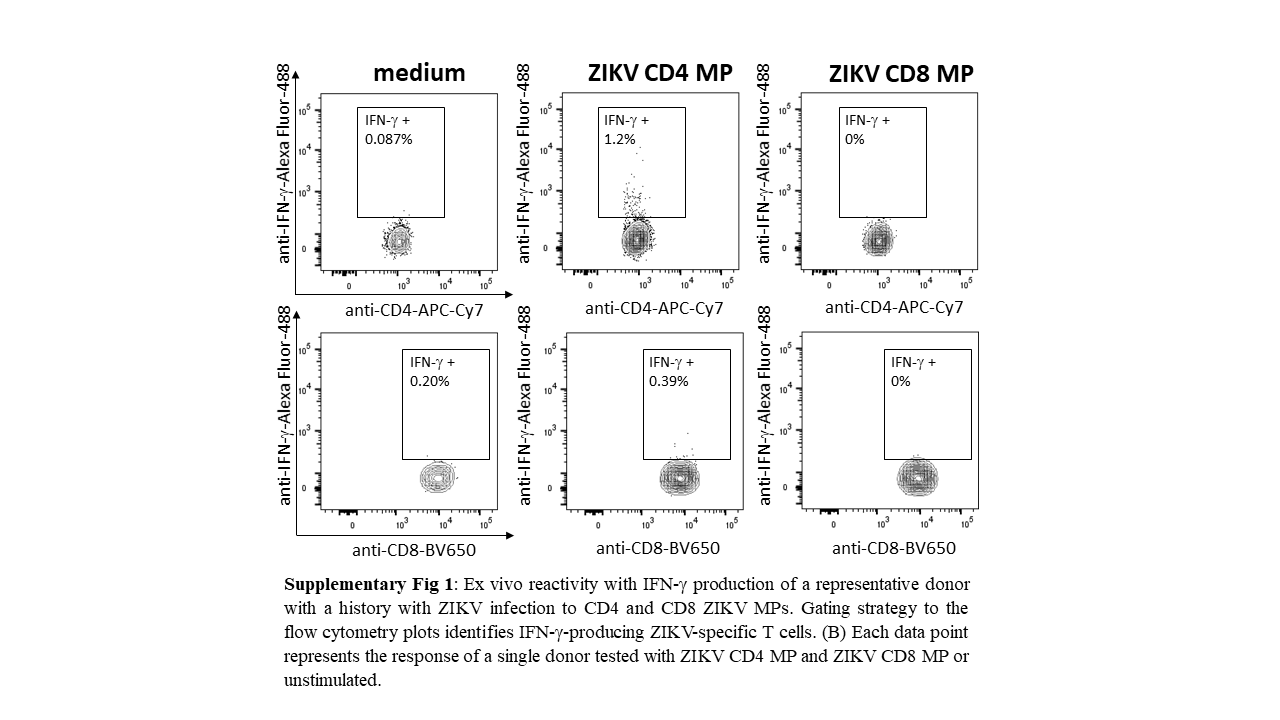

Supplement: Supplementary file 1 [file Image_1.TIF]
