# Supplementary material for: Differential Longevity of Memory CD4 and CD8 T Cells in a Cohort of the Mothers With a History of ZIKV Infection and Their Children
Source: Front Immunol. 2021 Feb 12;12:610456. doi: 10.3389/fimmu.2021.610456 (PMC7928292; doi:10.3389/fimmu.2021.610456)
Supplement: Supplementary file 2 [file Table_1.docx]

**Supporting information**

**Supplementary Table 1. Antibodies used in BD FACS ARIA IIu flow cytometer.**

| **Target Molecule** | **Clone** | **Company** | **Catalog #** |
| --- | --- | --- | --- |
| IFN-γ | 4S.B3 | eBioscience | 11-7319-82 |
| CCR7 | G043H7 | Biolegend | 353220 |
| CD3 | UCHT1 | BD Bioscience | 557943 |
| CD4 | RPA-T4 | eBioscience | 47004942 |
| CD45RA | HI100 | eBioscience | 48045842 |
| CD8 | RPA-T8 | Biolegend | 301092 |
| Live/dead |  | Molecular Probes | L34955 |
| CD107a | H4A3 | Biolegend | 328630 |
